# Fractionated stereotactic radiotherapy of brain metastases: results of a retrospective study

**DOI:** 10.1186/s13014-023-02277-6

**Published:** 2023-05-22

**Authors:** Isabella Gruber, Philipp Stark, Karin Weidner, Marius Treutwein, Oliver Koelbl

**Affiliations:** 1 Department of Radiation Oncology, University Hospital Regensburg, Franz-Josef-Strauss Allee 11, Regensburg, Bavarian, Germany; 2 University of Regensburg, Universitätsstraße 31, Regensburg, Bavarian, Germany

**Keywords:** Fractionated stereotactic radiotherapy, Brain metastases, Brain radiation necrosis, Malignant melanoma, Renal cell cancer

## Abstract

**Background:**

Lasting local control of brain metastases following stereotactic radiotherapy is becoming increasingly relevant since systemic treatment constantly improves the prognosis of patients with extracranial metastases.

**Methods:**

73 patients with 103 brain metastases received hypofractionated stereotactic radiotherapy (FSRT) in 6 fractions of 5 Gy between January 2017 and December 2021 at the University Hospital Regensburg, Germany. The study retrospectively evaluated local progression free survival (LPFS), overall survival (OS) and distant brain progression free survival (DPFS) of patients without prior radiotherapy of the brain. Response rate and brain radiation necrosis were reported. Cox proportional hazard models evaluated prognostic factors of OS and LPFS.

**Results:**

The median patient age was 61.0 years (Interquartile range, IQR 51.0, 67.5). The most common tumor types were malignant melanoma (34.2%) and non-small cell lung adenocarcinoma (26.0%). The median gross tumor volume (GTV) was 0.9 cm³ (IQR 0.4, 3.6). The median follow-up time of all patients was 36.3 months (95%CI 29.1, 43.4). The median OS was 17.4 months (95%CI 9.9, 24.9). Overall survival rates at 6-, 12-, 18-, 24-, and 30 months were 81.9%, 59.1%, 49.0%, 41.3%, and 37.2%, retrospectively. The mean LPFS was 38.1 months (95%CI 31.4, 44.9), while the median LPFS has not been reached. LPFS rates at 6-, 12-, 18-, 24- and 30 months were 78.9%, 68.7%, 64.3%, 61.6% and 58.7%, retrospectively. Median DPFS of all patients was 7.7 months (95%CI 6.1, 9.3). Six, 12-, 18-, 24- and 30 months DPFS rates were 62.1%, 36.3%, 31.1%, 24.8% and 21.7%. Five brain metastases (4.8%) developed brain radiation necrosis. In multivariate analysis, the number of brain metastases negatively affected LPFS. Non-melanoma and non-renal cell cancer was associated with a higher chance of LPFS in comparison to other cancer. A GTV > 1.5 cm³ translated into a higher risk of death compared to a GTV ≤ 1.5 cm³ and Karnofsky performance score was predictive of OS.

**Conclusions:**

FSRT in 6 fractions of 5 Gy seems to be an effective treatment with an acceptable local control for patients with brain metastases although melanoma and renal cell cancer seem to have a worse local control in comparison to other cancer.

**Trial registration:**

This study is retrospectively registered.

## Background

Brain metastases are common neoplasms in cancer patients [1, 2]. Given improvements in the systemic treatment and prolonged overall survival of patients with extracranial metastases, lasting control of irradiated brain metastases gains clinical significance, especially since many systemic agents have unclear CNS activity. Single fraction stereotactic radiosurgery (SRS) and hypofractionated stereotactic radiotherapy (FSRT) are both treatment modalities for patients with brain metastases [2]. SRS investigated in prospective studies is currently the international standard for local radiotherapy of brain metastases [3, 4] as the results of prospective randomized trials comparing the efficacy and safety of FSRT and SRS are lacking. However, data indicate that dose fractionation instead of SRS may yield in a better local tumor control by using a higher biologically effective dose (BED) [5]. Further advantage of dose fractionation can be the effect of reoxygenation of tumor cells between the fractions [6] and the reduced risk of brain radiation necrosis due to lower single doses [7, 8] compared to SRS. The objective of this retrospective study was to analyze our single-institution outcomes of patients with brain metastases following FSRT (6 × 5 Gy fractionation scheme).

## Methods

### Data collection

We retrospectively analyzed local control and overall survival of patients following FSRT of brain metastases at the Department of Radiation Oncology of the University Hospital Regensburg, Germany. Eligibility criteria for this retrospective analysis included patients with brain metastases of a solid cancer who received their first FSRT in 6 fractions of 5 Gy between January 2017 and December 2021. Patients who previously received radiotherapy of brain metastases (whole brain radiotherapy, SRS and/or FSRT) were excluded. All patients were reviewed at a multidisciplinary tumor conference. The choice of treatment of brain metastases (resection, radiotherapy) was dependent of size of brain metastases, neurological deficits due to brain edema, patient age, disease risk and/or presence of comorbidities. Clinical data were extracted from the medical charts of the University Hospital Regensburg, Germany. Variables included patient age at the time of FSRT, sex, diagnosis, initial UICC stage according to the TNM Classification of Malignant Tumours (8th edition), start date and end date of FSRT, gross tumor volume (GTV), planning target volume (PTV), location of brain metastases, control of primary cancer, presence of extracranial metastases, number of brain metastases treated with 6 × 5 Gy, Karnofsky performance score (KPS), recursive partitioning analysis (RPA), and diagnosis-specific graded prognostic assessment (ds-GPA). RPA and ds-GPA were calculated as defined by Gaspar et al. [9] and Sperduto et al. [10]. Age and KPS were assessed on the day of FSRT. Extracranial disease status and control of primary cancer related to the last medical evaluation before FSRT. Variables related to outcome were local progression free survival (LPFS), overall survival (OS), distant brain progression free survival (DPFS) and response of brain metastases on 1st follow-up magnetic resonance imaging (MRI; complete/partial response vs. stable disease vs. progressive disease). We performed a retrospective differentiation between progression, brain radiation necrosis and response of brain metastases based on follow-up imaging and/or histology in cases of resection. We analyzed cases of brain radiation necrosis and distinguished between radiographic changes of brain radiation necrosis without neurological symptoms and brain radiation necrosis resulting in neurological deficits. Data closing was January 2023. The local Ethics Board of the University of Regensburg approved this analysis (Ethics approval number: 22-2868-104). Consent for study participation was available in all patients due to BayKrG (Bayerisches Krankenhausgesetz) Art. 27 of the Bavarian legislation.

### Fractionated stereotactic radiotherapy and response assessment

All patients were immobilized with an individual stereotactic mask system of thermoplastic material (Brainlab, Munich, Germany) and received a planning computed tomogram (CT). CT slice thickness was 1 mm. Diagnostic contrast-enhanced T1-weighted MRIs were fused with CT scans. As an internal requirement, MRIs used were not allowed be older than 2 weeks. The gross tumor volume (GTV) was the demonstrable volume of the brain metastases as determined by MRI. The planning target volume (PTV) was created with a margin of 2–3 mm isotropic margin around the GTV. All brain metastases were treated with FSRT and a total dose of 30 Gy in 6 daily fractions of 5 Gy. The normalized dose (100% corresponding 30 Gy) was prescribed either to the mean value in the PTV (part I) or to the median dose (part II), while D0.03 cc < 33.0 Gy was accepted [11]. This change has been caused by the exchange of the treatment planning system, not allowing the same prescription technically and resulting in slight differences only. First we used Oncentra® external beam treatment planning system and collapsed cone algorithm for dose calculation from January 2017 to October 2018 (part I), and second Monaco® treatment planning system with Monte Carlo dose calculation from November 2018 to December 2021 (part II). Depending on the size and position of the brain metastases, patients received coplanar and non-coplanar 6 megavoltage (MV) photon beams with a linear accelerator of type Elekta Synergy™ or SynergyS™ (Elekta Ltd, Crawley, UK). FSRT was performed about three times a week excluding weekends. The BED was calculated using the linear-quadratic model. The BED was 42.5 Gy, assuming an alpha/beta of 12 Gy for brain metastases. We used kV X-ray/cone beam CT imaging for daily setup verification and repositioning.

Patients were followed-up with gadolinium-enhanced MRIs at 6–8 weeks after FSRT and every 3 months thereafter until the last follow-up appointment or until the date of death. We used modified definitions for response assessment of brain metastases similar to proposals from the Response Assessment in Neuro-Oncology Brain Metastases (RANO-BM) group [12]. Complete response and partial response were defined as disappearance of the irradiated brain metastasis in contrast-enhanced MRI and at least a 30% decrease in the sum longest diameter of the brain metastasis [12]. We summarized complete response and partial response as responsive disease. Progressive disease was defined as at least a 20% increase in the sum longest diameter of the brain metastasis and an increase by 5 mm or more. Stable disease was defined as neither fulfilling the criteria for progressive disease nor partial response [12]. In cases of radiographically assumption of progression, but clinical evidence assumes radiologically changes/brain radiation necrosis due to treatment effects and not to progression, MRIs were repeated in a shorter time interval, generally within about 6–8 weeks. Advanced imaging (perfusion MRI, PET-CT with amino acids) was not performed in each case and surgical resection only in symptomatic patients. Continued growth of the enhancing areas in follow-up imaging was considered as radiographically progression. Stable disease or regression of enhancing areas on serial follow-up MRIs were retrospectively considered as response. If repeated imaging or pathology showed response or progression of enhancing areas, the date of response or progression was recorded as the date of the initial scan [12].

### Definitions and statistical endpoints

The primary endpoint was LPFS. Secondary endpoints were OS, DPFS and response on 1st follow-up MRI. All times to the endpoints were calculated from the last day of FSRT. LPFS was defined as the time between the last day of FSRT and the first follow-up MRI showing in-field progression of the irradiated brain metastases. DPFS was defined as the time between the last day of FSRT and the appearance of distant brain failure (appearance of new or progressive brain metastases outside the PTV). OS was defined as the time from the last day of FSRT to the date of death by any cause. If a patient was event-free for all of the endpoints, the patient was censored at the last date of MRI or follow-up with confirmation of being event-free. OS and DPFS were calculated for all patients, whereas LPFS referred to the irradiated brain metastases. OS, LPFS and DPFS were evaluated using Kaplan-Meier estimators. Comparisons between groups were performed with Log-rank tests. Multivariable regression analyses were performed for OS and LPFS. Covariates were GTV, extracranial metastases, control of primary cancer, systemic treatment 3 months before/after FSRT, Karnofsky performance score, patient age, RPA, dsGPA, histology, cerebral progression outside of the PTV and number of brain metastases.

### Statistical analysis

Characteristics are presented as median and interquartile range (IQR) for continuous variables and as absolute and relative frequencies for categorical variables. OS and LPFS were analyzed by univariable and multivariable Cox proportional hazard regression models. Hazard Ratio (HR) and 95% - confidence interval (95% - CI) were presented as effect estimate. Median follow-up time was estimated by the reverse Kaplan-Meier method. All P - values were two-sided and P - values < 0.05 were considered significant. Statistical analysis was performed using SPSS 26.0 (SPSS Inc., Chicago, IL, USA).

## Results

### Patients and brain metastases characteristics

In total, 91 patients with 131 brain metastases received FSRT between January 2017 and December 2021. Eighteen patients previously treated with radiotherapy of brain metastases were excluded. In summary, 73 patients with 103 brain metastases were included in the analysis. Follow-up data were reported to January 2023. The median follow-up time of all patients was 36.3 months (95%CI 29.1, 43.4). The median time between the first day and the last day of FSRT were 9.0 days (IQR 8.0, 11.0). Patients` characteristics (n = 73) at the time of FSRT are summarized in Table 1.

**Table 1:** Characteristics of patients at the time of fractionated stereotactic radiotherapy (n = 73)

Characteristics	Value
Patient age, years, median (Interquartile range, IQR)	61.0 (51.0, 67.5)
Sex, n (%)	
Men	40 (54.8%)
Women	33 (45.2%)
Primary cancer, n (%)	
Malignant melanoma	25 (34.2%)
NSCLC adenocarcinoma	19 (26.0%)
NSCLC non-adenocarcinoma	7 (9.6%)
Breast cancer	6 (8.2%)
Gastrointestinal carcinoma	5 (6.8%)
Renal cell carcinoma	1 (1.4%)
Other	10 (13.7%)
Initial UICC stage, n (%)	
I	8 (10.9%)
II	8 (10.9%)
III	15 (20.5%)
IV	38 (52.0%)
Unknown/Missing	4 (5.5%)
Karnofsky performance score, median (IQR)	70 (65, 90)
Systemic treatment ^*^ 3 months before/after fractionated stereotactic radiotherapy, n (%)	66 (90.4%)
Number of brain metastases treated with 6x5Gy, median (IQR)	1.0 (1.0, 2.0)
Recursive partitioning analysis (RPA), median (IQR)	2.0 (2.0, 2.5)
Recursive partitioning analysis (RPA), RPA class, n (%)	
1	13 (17.8%)
2	42 (57.5%)
3	18 (24.7%)
Diagnosis-specific graded prognostic assessment (ds-GPA), median (IQR)	1.5 (1.0, 2.5)
Diagnosis-specific graded prognostic assessment (ds-GPA), groups	
0–1.0	21 (28.8%)
1.5–2.0	30 (41.1%)
2.5–3.0	21 (28.8%)
3.5–4.0	1 (1.4%)
Disease control of the primary cancer, n (%)	
Yes	53 (72.6%)
No	11 (15.1%)
Missing/Unknown	9 (12.3%)
Extracranial metastases, n (%)	
Yes	48 (65.8%)
No	25 (34.2%)

^*^ systemic treatment: chemotherapy, immunotherapy, targeted therapy or anti-hormonal therapy

IQR: interquartile range

Table 2 shows the characteristics of 103 brain metastases at the time of FSRT and 1st follow-up MRI. There were 21.8 months (IQR 9.6, 50.6) between diagnosis of primary cancer and FSRT of brain metastases. In the 1st follow-up MRI performed after a median follow-up of 48 days (IQR 39.0, 61.0), 53.4% of brain metastases showed responsive disease, 36.9% stable disease and 9.7% progressive disease, retrospectively. Five brain metastases (4.8%) showed brain radiation necrosis (Table 2).

**Table 2:** Characteristics of brain metastases treated with 6 × 5 Gy fractionated stereotactic radiotherapy (n = 103)

Characteristics	Value
Location, n (%)	
Supratentorial	81 (78.6%)
Infratentorial	22 (21.4%)
Gross tumor volume, GTV (cm³)	
Mean (Standard deviation, SD)	2.6 (4.5)
Median (Interquartile range, IQR)	0.9 (0.4, 3.6)
Planning target volume, PTV (cm³)	
Mean (SD)	7.5 (9.7)
Median (IQR)	3.4 (1.8, 9.3)
Response of the metastases on 1st follow-up MRI after a median time of 48 days (IQR 39.0, 61.0), n (%)	
Responsive disease *^,^†	55 (53.4%)
Stable disease ‡	38 (36.9%)
Progressive disease §	10 (9.7%)

SD: standard deviation; IQR: interquartile range; GTV: gross tumor volume, PTV: planning target volume, MRI: magnetic resonance imaging

* Complete response (disappearance of the brain metastasis on follow-up MRI) and partial response (at least a 30% decrease in the sum longest diameter of the brain metastasis) were summarized as responsive disease

† Including 5 brain metastases of 4 patients with brain radiation necrosis: Four brain metastases showed radiographically signs of brain radiation necrosis (patients had no neurological symptoms) and regression of enhancing areas on serial follow-up MRIs. There was 1 biopsy proven brain radiation necrosis of a brain metastasis of a patient with neurologic deficits. Histology showed no residual tumor cells. All 4 patients with brain radiation necrosis (4 x malignant melanoma, 1 x adrenal cancer) received immunotherapy at the time of diagnosis of brain radiation necrosis

‡ Stable disease was defined as neither fulfilling the criteria for partial response nor progressive disease

§ Progressive disease was defined as at least a 20% increase in the sum longest diameter of the brain metastasis and an increase by 5 mm or more

### Overall survival

**Fig. 1:**
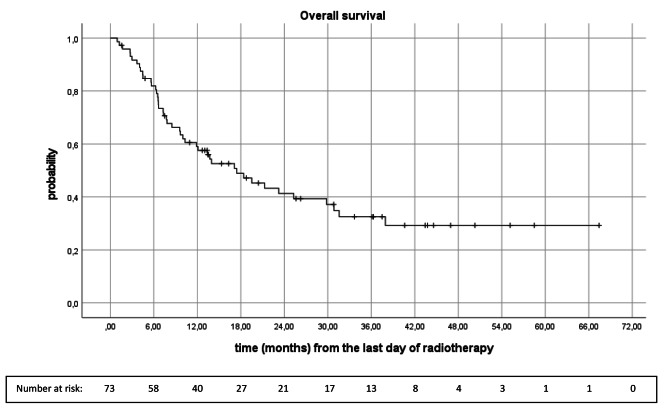
Kaplan-Meier analysis of overall survival following radiotherapy of brain metastases (n = 73)

Median OS was 17.4 months (95%CI 9.9, 24.9). Overall survival rates at 6-, 12-, 18-, 24-, and 30 months were 81.9%, 59.1%, 49.0%, 41.3%, and 37.2%, retrospectively (Fig. 1).

**Fig. 2:**
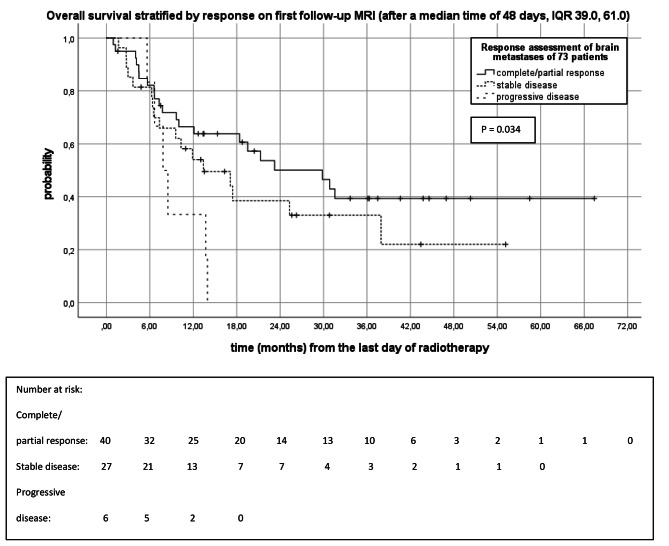
Kaplan-Meier analysis of OS stratified by response on 1st follow-up MRIs (n = 73) In cases of patients with > 1 brain metastases, the worst response was analyzed and progression was defined as at least one brain metastasis fulfilling the criteria of progression. Complete response (disappearance of the brain metastasis) and partial response (at least a 30% decrease in the sum longest diameter of the brain metastasis) in MRI were summarized as response. Stable disease was defined as neither fulfilling the criteria for partial response nor progressive disease. Progressive disease was defined as at least a 20% increase in the sum longest diameter of the brain metastasis and an increase by 5 mm or more in MRI

Figure 2 depicts the Kaplan-Meier analysis of OS stratified by response on 1st follow-up MRIs, which were performed after a median time of 48 days (IQR 39.0, 61.0). The median OS of patients with complete/partial response on 1st follow-up MRI was 29.8 months (95%CI 16.3, 43.3), retrospectively (Fig. 2). The median OS of patients with stable disease and progressive disease on 1st follow-up MRI were 13.4 months (95%CI 4.0, 22.8) and 7.8 months (95%CI 5.6, 10.0), retrospectively (P = 0.034). Patients with complete/partial response on 1st follow-up MRI had 6-, 12-, 18-, 24- and 30 months OS rates of 82.2%, 66.5%, 63.8%, 50.1% and 46.5%. Patients with stable disease on 1st follow-up MRI had a 6-, 12-, 18-, 24- and 30 months OS of 81.5%, 54.0%, 38.5%, 38.5%, and 33.0%. Patients with progressive disease had a 6-, 12- and 18-months OS of 83.3%, 33.3%, and 0%.

### Local progression free survival

**Fig. 3:**
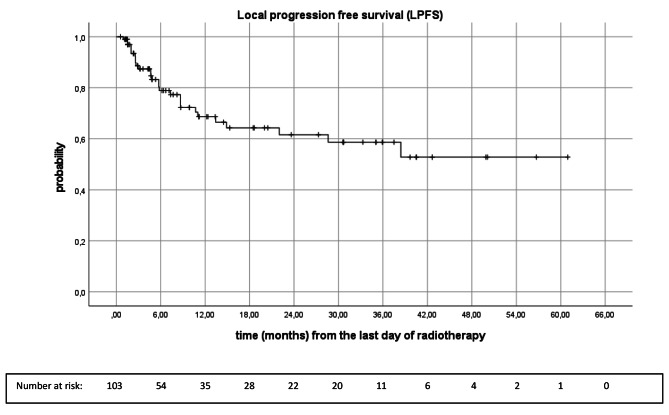
Kaplan-Meier analysis of local progression free survival of brain metastases (n = 103)

Mean LPFS was 38.1 months (95%CI 31.4, 44.9), while the median LPFS has not been reached. Local progression free survival rates at 6-, 12-, 18-, 24- and 30 months were 78.9%, 68.7%, 64.3%, 61.6% and 58.7%, retrospectively (Fig. 3).

**Fig. 4:**
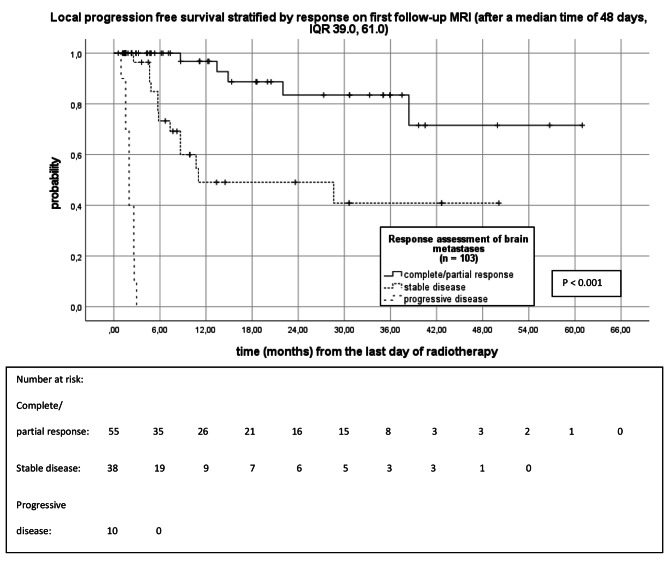
Kaplan-Meier analysis of LPFS stratified by response on 1st follow-up MRI (n = 103) Complete response (disappearance of the brain metastasis) and partial response (at least a 30% decrease in the sum longest diameter of the brain metastasis) were summarized as response. Stable disease was defined as neither fulfilling the criteria for partial response nor progressive disease. Progressive disease was defined as at least a 20% increase in the sum longest diameter of the brain metastasis and an increase by 5 mm or more.

Figure 4 shows the Kaplan-Meier analysis of LPFS of brain metastases stratified by response on 1st follow-up MRIs, which were performed after a median time of 48 days (IQR 39.0, 61.0). The median LPFS of brain metastases with complete/partial response on 1st follow-up MRI has not been reached. Mean LPFS of brain metastases with complete/partial response on 1st follow-up MRI was 50.8 months (95%CI 42.8, 58.7). Median LPFS of brain metastases with stable and progressive disease on 1st follow-up MRI were 11.0 months (95%CI 0.0, 32.9) and 2.0 months (95%CI 1.5, 2.5), retrospectively (P < 0.001). Brain metastases showing complete/partial response on 1st follow-up MRI had a 6-, 12-, 18-, 24- and 30 months LPFS of 100%, 96.8%, 88.7%, 83.5%, and 83.5%. Brain metastases with stable disease on 1st follow-up MRI had a 6-, 12-, 18-, 24-, and 30 months LPFS of 73.3%, 49.1%, 49.1%, 49.1%, and 40.9% (Fig. 4).

### Distant progression free survival

**Fig. 5:**
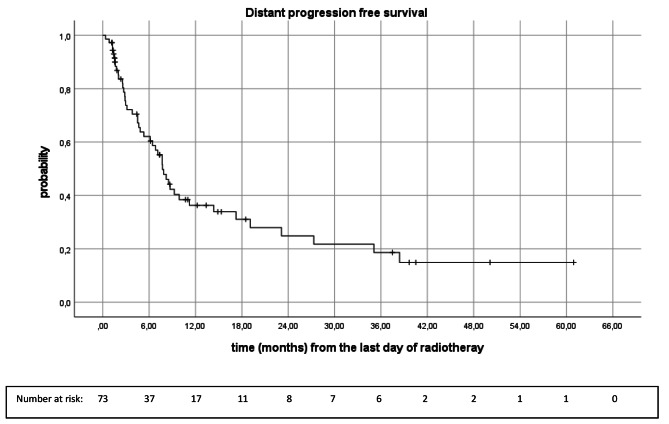
Kaplan-Meier analysis of distant progression free survival (n = 73)

Figure 5 shows the Kaplan-Meier analysis of DPFS (n = 73). Median DPFS was 7.7 months (95%CI 6.1, 9.3). Six, 12-, 18-, 24- and 30 months DPFS rates were 62.1%, 36.3%, 31.1%, 24.8% and 21.7%, retrospectively (Fig. 5). In total, 61.6% (n = 45) of patients had brain distant relapse. In cases of brain distant relapse, 31 patients (68.9%) received another course of stereotactic radiotherapy. Two patients (4.4%) received whole brain radiotherapy, 3 patients (6.7%) resection of brain metastases, 2 patients (4.4%) beginning of immunotherapy and 7 patients (15.5%) best supportive care.

### Univariate and multivariate analysis of OS and LPFS

Results of the univariate and multivariate analysis of OS are shown in Table 3. In multivariate analysis, the size of GTV was strongly associated with OS. A GTV > 1.5cm³ translated into a higher risk of death compared to a GTV ≤ 1.5cm³ (HR 3.620, 95%CI 1.413, 9.273; P = 0.007). KPS was predictive of OS (HR 0.941, 95%CI 0.889, 0.996; P = 0.034). The presence of extracranial metastases, RPA and ds-GPA lost significance in the multivariate model (Table 3).

**Table 3:** Univariate and multivariable analysis of overall survival (n = 73)

	Univariate model			Multivariate model		
Characteristics	HR	95%CI	P - value	HR	95%CI	P - value
Gross tumor volume (cm³)						
≤ 1.5cm³ (reference)						
> 1.5cm³	2.301	1.233, 4.294	**0.009**	3.620	1.413, 9.273	**0.007**
Extracranial metastases						
No (reference)						
Yes	3.627	1.659, 7.933	**0.001**	0.908	0.209, 3.946	0.898
Control of primary cancer						
Control (reference)						
No control	1.385	0.608, 3.157	0.438	1.330	0.526, 3.360	0.547
Systemic treatment * 3 months before/after FSRT						
Yes (reference)						
No	1.632	0.633, 4.208	0.310	1.779	0.450, 7.029	0.412
Karnofsky performance score (KPS)	0.949	0.924, 0.975	**< 0.001**	0.941	0.889, 0.996	**0.034**
Number of brain metastases	1.121	0.832, 1.509	0.453	1.091	0.655, 1.819	0.737
Patient age	1.012	0.988, 1.037	0.334	0.967	0.920, 1.016	0.187
Recursive partitioning analysis (RPA)	2.506	1.550, 4.051	**< 0.001**	0.691	0.249, 1.921	0.479
Diagnosis-specific graded prognostic assessment (ds-GPA)	0.479	0.328, 0.700	**< 0.001**	0.543	0.155, 1.910	0.341
Histology						
Malignant melanoma/renal cell cancer (reference)						
Other	1.127	0.608, 2.089	0.704	0.482	0.200, 1.160	0.103
Cerebral progression outside the planning target volume						
No (reference)						
Yes	1.356	0.708, 2.594	0.358	1.574	0.692, 3.582	0.279

* systemic treatment: chemotherapy, immunotherapy, targeted therapy or anti-hormonal therapy; FSRT: fractionated stereotactic radiotherapy

Table 4 shows the results of the univariate and multivariate analysis of LPFS. In multivariate analysis the number of brain metastases negatively affected LPFS (HR 2.353, 95%CI 1.374, 4.028; P = 0.002). Histology of a non-melanoma and non-renal cell cancer was associated with a higher chance of LPFS in comparison to melanoma and renal cell histology (HR 0.155, 95%CI 0.049, 0.492; P = 0.002)

**Table 4:** Univariate and multivariate model of local progression free survival (n = 103)

	Univariate model			Multivariate model		
Characteristics	HR	95%CI	P - value	HR	95%CI	P - value
Gross tumor volume (cm³)						
≤ 1.5cm³ (reference)						
> 1.5cm³	1.016	0.442, 2.336	0.969	2.063	0.717, 5.933	0.179
Extracranial metastases						
No (reference)						
Yes	4.141	1.500, 11.430	**0.006**	4.504	0.752, 26.966	0.099
Control of primary cancer						
Control (reference)						
No control	0.955	0.287, 3.180	0.940	2.278	0.534, 9.716	0.266
Karnofsky performance score (KPS)	0.986	0.957, 1.016	0.358	0.985	0.921, 1.053	0.650
Systemic treatment * 3 months before/after FSRT						
Yes (reference)						
No	1.551	0.582, 4.134	0.380	0.956	0.299, 3.057	0.939
Number of brain metastases	1.699	1.232, 2.343	**0.001**	2.353	1.374, 4.028	**0.002**
Patient age	1.007	0.978, 1.036	0.648	1.067	0.998, 1.142	0.059
Recursive partitioning analysis (RPA)	1.275	0.691, 2.352	0.437	1.182	0.615, 2.273	0.616
Diagnosis-specific graded prognostic assessment (dsGPA)	0.584	0.373, 0.916	**0.019**	2.386	0.495, 11.491	0.278
Histology						
Malignant melanoma/renal cell cancer (reference)						
Other	0.500	0.233, 1.072	0.075	0.155	0.049, 0.492	**0.002**
Cerebral progression outside the planning target volume						
No (reference)						
Yes	0.830	0.381, 1.807	0.639	1.508	0.566, 4.018	0.411

* systemic treatment: chemotherapy, immunotherapy, targeted therapy or anti-hormonal therapy; FSRT: fractionated stereotactic radiotherapy

## Discussion

The retrospective study analyzed a single-institutional experience in FSRT of brain metastases with 6 fractions of 5 Gy. Literature shows various dose schedules for FSRT of brain metastases. Unfortunately, there are no guidelines for the number of fractions and single doses to use in FSRT of brain metastases, and the choice of fractionation seems to be individual according to the experience of each center. Our data revealed a median OS of 17.4 months (95%CI 9.9, 24.9). Six- and 12 months OS rates were 81.9% and 59.1%, retrospectively. Similar median OS and 6- and 12 months OS rates were reported by Fahrig et al. [13]. Wiggenraad et al. [5] described a dose-response relationship between BED and local control of brain metastases at 12 months and demanded a BED 12 (using an alpha/beta value of 12 Gy for brain metastases) of at least 40 Gy to obtain a 12 month local control rate of ≥ 70%. The median LPFS of our study population has not been reached. Mean LPFS was 38.1 months (95%CI 31.4, 44.9) and 6-, 12- and 24 months LPFS rates were 78.9%, 68.7% and 61.6%, retrospectively. Other studies yielded similar 12 months LPFS rates. Narayana et al. [14] reported outcomes of 20 patients following FSRT with 5 × 6 Gy prescribed to the 100% isodose line encompassing the PTV. The median follow-up was 10 months (range 1–18) and the 1-year local control rate was 70%. The median OS was 8.5 months and the 1-year OS rate was 42% [14]. Kim et al. [15] reported data of 40 patients treated with 6 × 6 Gy FSRT in comparison to SRS. Patients of the FSRT group had 6- and 12 months LPFS rates of 97% and 69%, and 6- and 12 months OS rates of 60% and 31%, retrospectively [15]. Similar LPFS was observed by de la Pinta et al. [16] using 30 Gy in 5–6 fractions. LPFS rates at 6- and 12 months were 80% and 69%, retrospectively [16]. Slightly better LPFS rates were reported by Fokas et al. [17]. Fokas et al. [17] retrospectively evaluated the use of two different FSRT regimen (10 × 4 Gy, n = 61 and 7 × 5 Gy, n = 61) in comparison to SRS. The 6- and 12- months LPFS rates were 87% and 75% for the 7 × 5 Gy group and 81% and 71% for the 10 × 4 Gy group [17]. The median OS were 7 months for patients treated with 7 × 5 Gy, and 10 months for patients treated with 10 × 4 Gy, retrospectively. A recent study on different dose schedules of 41 patients revealed that doses lower than 30 Gy in 5 fractions were associated with a lower local control [18]. The authors therefore support the use of 7–8 Gy in five fraction stereotactic radiotherapy [18]. A recent study containing melanoma and renal cell cancer and using different fractionation schemes (most used schedules: 3 × 10 Gy, 6 × 6 Gy or 3 × 9 Gy) confirms our results [19]. Lesueur et al. [19] reported LPFS rates of 72% and 68% at 12- and 18 months after a median follow-up of 7.4 months. As demonstrated above the findings regarding local control and OS following FSRT vary. At this point we should also note the fact that many of these studies mentioned are confounded by differing prescription doses and fractionation schemes and some have major biases such as small patient numbers. In some studies follow-up may be too short to detect relapses. More frequent follow-up imaging detects recurrences or new metastases earlier than longer periods between follow-ups. Moreover, the use of different response criteria of brain metastases (uni- or bi-dimensional evaluation or volumetric evaluation) and mixed cancer entities are other topics of bias.

We performed the 1st follow-up MRIs after a median time of 48 days, retrospectively. Our data indicate that response of brain metastases in the 1st follow-up MRI seems to be predictive for OS and LPFS, although there are certainly brain metastases with delayed response following FSRT. A potential treatment effect is brain radiation necrosis. Brain radiation necrosis (4.8%) was relatively low in our study. Interestingly, all patients with brain radiation necrosis had ongoing immunotherapy at the time of diagnosis of brain radiation necrosis. It is challenging to distinguish between brain radiation necrosis and true progression/local recurrence. The diagnosis of brain radiation necrosis is mostly based on radiographically findings since biopsies or resections are only performed in cases with disabling neurological symptoms related to cerebral edema. In most cases, contrast enhancement and reactive edema resolve without change of treatment. The uncertainty of distinguishing between recurrence and brain radiation necrosis should be taken into account when considering studies that do not provide information about brain radiation necrosis. A recent study of Lesueur et al. [19] analyzed results following FSRT of brain metastases from melanoma and renal cancer and reported radionecrosis in 7.1% of brain metastases following FSRT. In summary, our rate of brain radiation necrosis was similar to other studies using FSRT [20, 19]. A third of our patients had brain metastases of malignant melanoma or renal cell cancer. Both histological cancer types showed worse local control in comparison to other histological types. A similar trend was observed by Minniti et al. [20]. Our multivariate analysis revealed that the size of GTV was strongly associated with OS. This could be a function of intrinsic radiation resistance of larger brain metastases. The predictive value of the size of GTV is in line to the findings of other groups [21]. KPS is a known prognostic variable correlated to OS [22, 20, 21]. Several studies on prognostic indices concluded that the number of brain metastases correlates with OS [23, 22]. Our data indicate a predictive function of the number of brain metastases for LPFS but not for OS. One possible reason for the lack of influence on OS may be the relatively favorable OS of our patient cohort and the relatively small GTVs. It is worth to note, that older patients responded as well as younger patients [22]. Although some studies have demonstrated that dsGPA [20] and RPA [17, 13] could have prognostic value, both variables did not significantly affect OS or LPFS on multivariate analyses. Regarding the variables extracranial metastases and activity of primary cancer we have to note that the detection of both requires comprehensive imaging such as PET-CT and PET-imaging was not available in all patients.

DPFS after FSRT seems to be poor necessitating further therapy. In our study, 68.9% of patients had received a second session of stereotactic radiotherapy of new brain metastases avoiding whole brain radiotherapy in most of patients. Due to the focus on a single center there is the possibility of selection bias. Moreover, this study is limited by its retrospective nature and the relatively small number of patients. We acknowledge that the heterogeneity of cancer types may result in biases. Nevertheless, the study provides a useful and important insight into the treatment of brain metastases with FSRT. The primary strength of the study is the consistent delivery of FSRT and the length of follow-up. Additionally, data completeness was 100% through active monitoring of all patients. Although lesion size and proximity to critical structures remain critical to the choice of fractionation, it is still being discussed whether fractionation or not is the best treatment modality for patients with brain metastases. The prospective multicenter FSRT-Trial compares FSRT (12 × 4 Gy, prescription to the 80% isodose line encompassing the PTV) and SRS (according to RTOG 9005) in brain metastases (1–4 cm). Randomization is based on metastasis size and histology. The results of this study may help to improve local control of brain metastases following stereotactic radiotherapy finding the more effective treatment scheme.

## Conclusions

In summary, 30 Gy FSRT in 6 fractions appears to be an effective treatment modality for patients with brain metastases resulting in an acceptable local control although melanoma and renal cell cancer histology seem to be a risk for worse local control in comparison to other histologic types.

## Data Availability

The datasets generated and/or analyzed during the current study are not publicly available due to privacy but are available from the corresponding author on reasonable request.

## List of Abbreviations

BayKrG: Bayerisches Krankenhausgesetz
BED: Biologically effective dose
CI: Confidence intervals
CNS: Central nervous system
CT: Computed tomogram
DPFS: Distant progression free survival
ds-GPA: Diagnosis-specific graded prognostic assessment
FSRT: Fractionated stereotactic radiotherapy
GTV: Gross tumor volume
HR: Hazard ratio
IQR: Interquartile range
KPS: Karnofsky performance score
LPFS: Local progression free survival
MRI: Magnetic resonance imaging
MV: Megavoltage
OS: Overall survival
PET: Positron emission tomography
PTV: Planning target volume
RANO-BM: Response Assessment in Neuro-Oncology Brain Metastases
RPA: Recursive partitioning analysis
SRS: Stereotactic radiosurgery
